# Circulating Adiponectin and Its Association with Metabolic Traits and Type 2 Diabetes: Gene-Diet Interactions Focusing on Selected Gene Variants and at the Genome-Wide Level in High-Cardiovascular Risk Mediterranean Subjects

**DOI:** 10.3390/nu13020541

**Published:** 2021-02-07

**Authors:** Oscar Coltell, Carolina Ortega-Azorín, Jose V. Sorlí, Olga Portolés, Eva M. Asensio, Carmen Saiz, Rocío Barragán, Ramon Estruch, Dolores Corella

**Affiliations:** 1 Department of Computer Languages and Systems, Universitat Jaume I, 12071 Castellón, Spain; oscar.coltell@uji.es; 2 CIBER Fisiopatología de la Obesidad y Nutrición, Instituto de Salud Carlos III, 28029 Madrid, Spain; carolina.ortega@uv.es (C.O.-A.); jose.sorli@uv.es (J.V.S.); olga.portoles@uv.es (O.P.); eva.m.asensio@uv.es (E.M.A.); carmen.saiz@uv.es (C.S.); rocio.barragan@uv.es (R.B.); restruch@clinic.cat (R.E.); 3 Department of Preventive Medicine and Public Health, School of Medicine, University of Valencia, 46010 Valencia, Spain; 4 Sleep Center of Excellence, Department of Medicine, Columbia University Irving Medical Center, New York, NY 10032, USA; 5 Division of General Medicine, Department of Medicine, Columbia University Irving Medical Center, New York, NY 10032, USA; 6 Department of Internal Medicine, Hospital Clinic, Institut d’Investigació Biomèdica August Pi i Sunyer (IDIBAPS), University of Barcelona, Villarroel, 170, 08036 Barcelona, Spain

**Keywords:** adiponectin, type 2 diabetes, plasma lipids, body mass index, genetics, Mediterranean diet, polymorphisms, genome-wide association study, gene–diet interactions

## Abstract

Adiponectin is gaining renewed interest since, in addition to its possible protective role against insulin resistance and arteriosclerosis, recent studies suggest other additional favorable effects. However, the influence of gene-diet interactions on plasma adiponectin levels is still little understood. We analyzed the association between plasma adiponectin levels and various metabolic traits in a high-cardiovascular risk Mediterranean population, as well as the genetic effect of four candidate single-nucleotide polymorphisms (SNPs) in the adiponectin gene (*ADIPOQ*) and their interactions with the Mediterranean dietary pattern. Additionally, we explored, at the genome-wide level, the SNPs most associated with plasma adiponectin levels, as well as gene–diet interactions with the Mediterranean diet. In the 954 participants studied (aged 55–80 years), plasma adiponectin levels were strongly associated with plasma HDL-C concentrations (*p* = 6.6 × 10^−36^) and inversely related to triglycerides (*p* = 4.7 × 10^−18^), fasting glucose (*p* = 3.5 × 10^−16^) and type 2 diabetes (*p* = 1.4 × 10^−7^). Of the four pre-selected *ADIPOQ* candidate SNPs, the one most associated with plasma adiponectin was the −11391G > A (rs17300539) promoter SNP (*p* = 7.2 × 10^−5^, in the multivariable adjusted model). No significant interactions with the Mediterranean diet pattern were observed for these SNPs. Additionally, in the exploratory genome-wide association study (GWAS), we found new SNPs associated with adiponectin concentrations at the suggestive genome-wide level (*p* < 1 × 10^−5^) for the whole population, including the lead SNP rs9738548 (intergenic) and rs11647294 in the *VAT1L* (Vesicle Amine Transport 1 Like) gene. We also found other promising SNPs on exploring different strata such as men, women, diabetics and non-diabetics (*p* = 3.5 × 10^−8^ for rs2850066). Similarly, we explored gene–Mediterranean diet interactions at the GWAS level and identified several SNPs with gene–diet interactions at *p* < 1 × 10^−5^. A remarkable gene–diet interaction was revealed for the rs2917570 SNP in the *OPCML* (Opioid Binding Protein/Cell Adhesion Molecule Like) gene, previously reported to be associated with adiponectin levels in some populations. Our results suggest that, in this high-cardiovascular risk Mediterranean population, and even though adiponectin is favorably associated with metabolic traits and lower type 2 diabetes, the gene variants more associated with adiponectin may be population-specific, and some suggestive gene–Mediterranean diet interactions were detected.

## 1. Introduction

Since the discovery of adiponectin in 1995 [1], this hormone, mainly secreted by the adipose tissue and initially called adipocyte complement-related protein of 30 kDa (Acrp30), has drawn a great deal of attention due to its possible protective effect against type 2 diabetes, its anti-inflammatory effects and its anti-atherogenic properties [2,3,4,5]. It has also aroused controversy, given that although various studies reported that high plasma adiponectin levels were associated with lower cardiovascular disease risk [6,7,8,9], others were unable to confirm its protective effect against cardiovascular diseases or total mortality [10,11,12].

Given the interest that adiponectin has aroused, there is a large number of studies that have analyzed its associations with various adiposity parameters, insulin resistance, plasma lipid levels, inflammatory markers, type 2 diabetes risk and atherosclerosis biomarkers [13,14,15,16,17,18]. Although the results of several studies may differ, there is strong consistency in observing that adiponectin appears to increase insulin sensitivity by improving glucose and lipid metabolisms. Despite the fact that several studies have indicated that different foods or dietary patterns may increase plasma adiponectin levels [19,20,21,22,23,24,25,26], the truth is that there is scarce evidence on the role of the various dietary components on adiponectinemia. Moreover, adiponectin levels may have an important genetic component [27,28,29,30,31].

Although various candidate genes have been studied [29,32,33] and several genome-wide association studies (GWASs) have been undertaken [34,35,36,37,38,39,40,41,42,43,44,45], the polymorphisms in the main genes identified so far only account for 5% of the plasma adiponectin variation, leaving many genes yet to be identified. The leading candidate gene for adiponectin levels is the *ADIPOQ* (adiponectin) gene on chromosome 3 [32]. This gene has been identified as the main signal in several GWASs carried out in European populations or in white Caucasians [35,41,42,43,44,45]. However, in Asian populations, the strongest signal statistically associated with adiponectin levels is located in the *CDH13* (T-cadherin) gene, chromosome 16 [34,36,37,38,39,40,41]. Besides these signals, and depending on the population in which the study was undertaken, other top-ranked single-nucleotide polymorphisms (SNPs) in genes such as *TRIB1* (Tribbles Pseudokinase), *DNAH10* (Dynein heavy chain 10, axonemal) and *PEPD* (Peptidase D) were identified that reached a high level of statistical significance, but which differed greatly depending on the populations, therefore being highly inconsistent [35,36,37,38,39,40,41,42,43,44,45].

Even in a large GWAS meta-analysis, including more than 45,000 participants [41,44], it was not possible to strongly identify, at the GWAS level, a greater number of novel genes and gene variants associated with plasma adiponectin levels. That would suggest that, besides the possible ethnic differences depending on the geographical origin of the population, other environmental modulations such as diet and other lifestyle variables, as well as sex, age, obesity and type 2 diabetes, among others, may have had an influence on the results of the GWAS. Furthermore, most studies on candidate genes [46,47,48,49,50], as well as all the GWASs [35,36,37,38,39,40,41,42,43,44,45], focused on studying the genetic contribution without taking modulation by diet into account. Only very few studies focusing on candidate genes analyzed gene-diet interactions for adiponectin concentrations [51,52,53,54]. It is, therefore, essential to increase knowledge on the main gene–diet interactions determining both the plasma adiponectin levels at the candidate gene and genome-wide levels. Moreover, bearing in mind that the Mediterranean population has scarcely been included in adiponectin GWASs and that the Mediterranean diet can modulate genetic influence through different gene-diet interactions [55], our aims were as follows:

(1) To study the association between four pre-selected candidate gene variants: −11391 G/A (rs17300539); +45T > G (rs2241766); +276G > T (rs1501299); and rs17366568 (G > A), in the *ADIPOQ* gene on adiponectin levels and related traits (plasma lipids, adiposity and type 2 diabetes), as the main effect, and to analyze the gene-diet interactions with the Mediterranean dietary pattern in a high-cardiovascular risk Mediterranean population. (2) To undertake an exploratory GWAS to scan the gene variants most associated with plasma adiponectin levels in this population. Finally, (3) to explore, at the genome-wide level, the potential gene-Mediterranean diet interactions determining plasma adiponectin concentrations in this population.

## 2. Materials and Methods

### 2.1. Study Design and Participants

We carried out a cross-sectional study on 954 elderly high-cardiovascular risk Mediterranean subjects. These white subjects of European ancestry were recruited in the PREDIMED (Prevención con Dieta Mediterránea)-Valencia study [56]. The Valencia field center is located on the Mediterranean coast of the Iberian Peninsula (Spain). The subjects included in this study were those who had plasma adiponectin determination available at the baseline visit. These participants were recruited in primary health care centers, the inclusion criteria being as follows: elderly (between 55 and 80 years old for men and between 60 and 80 years old for women) and having a high cardiovascular risk, even though they were free of cardiovascular disease at baseline. Eligible subjects fulfilled at least one of the two criteria: type 2 diabetes; three or more of the following cardiovascular risk factors: current smoking; hypertension; dyslipidemia; body mass index (BMI) ≥ 25 kg/m^2^; and/or family history of premature cardiovascular disease [57,58]. These participants were recruited in the various primary health care centers of the Valencia region. Participants provided written informed consent and study protocols and procedures were approved according to the ethical standards of the Helsinki Declaration and by the Human Research Ethics Committee of Valencia University, Valencia (ethical approval code H1422226460525).

### 2.2. Baseline Anthropometric, Clinical, Biochemical and Lifestyle Variables

Anthropometric variables and blood pressure were determined at baseline by trained staff. Weight and height were measured with light clothing and no shoes with calibrated scales and a wall-mounted stadiometer, respectively, as previously reported. BMI was calculated as the weight (in kg) divided by the height (in m^2^). Obesity was defined as a BMI ≥ 30 kg/m^2^. Waist circumference was measured midway between the lowest rib and the iliac crest, after normal exhalation, using an anthropometric tape [56,58]. Further, in the baseline examination, we assessed demographic factors, cardiovascular risk factors, medications and lifestyle variables by validated questionnaires [58,59]. Type 2 diabetes was defined according to the criteria of the American Diabetes Association-2018, as previously reported [57,58]. Blood pressure was measured by trained personnel using a validated semi-automatic oscillometer (Omron HEM-70CP; Hoofddorp, The Netherlands) with the subject seated. Physical activity was estimated by the validated Minnesota Leisure-Time Physical Activity questionnaire as previously reported [56,57,58]. Smoking status was assessed by the World Health Organization questionnaire including five categories as previously reported [56,57,58,59]. Here, non-smokers and ex-smokers were combined and compared with current smokers. Thus, a dichotomic variable was used for smoking status.

As dietary variables for this study, we focused on the Mediterranean diet pattern. To measure the Mediterranean diet pattern, we used a validated 14-item scale [60], also administered at baseline. Detailed items of that scale with their response options are presented in Supplemental Table S1. Each question was scored 0 or 1. The final score ranged from 0 to 14. The higher the score, the greater the adherence to the Mediterranean diet. The degree of adherence was later dichotomized into high (≥9) and low (<9) depending on the population mean (9 points).

### 2.3. Biochemical Determinations and Plasma Adiponectin Measures

Blood samples were collected after a 12-h overnight fast. Fasting glucose, total cholesterol, triglycerides and HDL cholesterol (HDL-C) were measured using standard enzymatic automated methods as previously described [56,58]. In patients whose triglyceride levels were <400 mg/dL, LDL-C concentrations were estimated using the Friedewald formula. In addition, fasting blood samples were obtained for each participant and plasma was stored at −80 °C until further biochemical analyses. Fasting plasma adiponectin concentrations were measured and determined using an enzyme-linked immunosorbent assay (ELISA) kit (Cat. # EZHADP-61K), supplied by Linco Research, St. Charles, MO, USA, according to the manufacturer’s instructions. The absorbance was read at 450 nm using the Multiskan EX spectrophotometer (Thermo Electron Corporation, Milford, NH, USA) and the Ascent Software program (Thermo Labsystems, Vantaa, Finland). All determinations were made in duplicate. Adiponectin concentrations were expressed in µg/mL. The intra-assay coefficient of variation was 1.2% and that of the inter-assay was 2.1%.

### 2.4. DNA Isolation and Genotyping

Genomic DNA was isolated from blood. The quantity of double-stranded DNA was measured using PicoGreen (Invitrogen Corporation, Carlsbad, CA, USA). Firstly, we selected the relevant polymorphisms in the adiponectin gene (*ADIPOQ*) and undertook their specific genotyping. The four polymorphisms selected were initially the most commonly studied: a promoter variant, −11391 G/A (rs17300539) [49]; the T to G substitution in exon 2 (+45T > G, rs2241766); the G to T substitution in intron 2 (+276G > T, rs1501299) [47,48,49]; and rs17366568 (G > A) in intron 3 [35]. The SNPs (rs17300539 and rs2241766) in the *ADIPOQ* gene were determined by polymerase chain reaction-restriction fragment length polymorphism (PCR-RFLP) [61] using MspI and SmaI, respectively. TaqMan real-time PCR using pre-designed assays for allelic discrimination, containing specific probes for each allele (ThermoFisher Scientific, Waltham, MA, USA), was used for the rs1501299 and the rs17366568 genotyping (C_7497299_10 and C_33187752_10, respectively) on a 7900HT Sequence Detection system (Applied Biosystems, Foster City, CA, USA) [20]. Quality control measures of SNP genotyping were carried out including replicate quality control samples (10%) and no deviation from the Hardy-Weinberg equilibrium testing.

Besides the genotyping of these pre-selected polymorphisms in the *ADIPOQ* gene, a high-density genotyping at the genome-wide level using the Infinium OmniExpress-24 BeadChip genotyping array (v1.0 and v1.1) (Illumina Inc., San Diego, CA, USA) was then carried out. This array captures approximately 720,000 markers (the number varies depending on the version used: 730,000 for v.1.0 and 716,000 for v.1.1), and 699,221 markers that are common to both versions of the array (v1.0 and v.1.1) were used. Genome-wide genotyping was performed at the University of Valencia according to the manufacturer’s protocol with appropriate quality standards as previously reported [56]. Allele detection and genotype calling were performed in the GenomeStudio genotyping module (Illumina, Inc.). Data cleaning was performed using standard analysis pipelines implemented in the Phyton programming language combined with PLINK [62,63]. We filtered out the SNPs not mapped on autosomal chromosomes. In addition, SNPs with a minor allele frequency (MAF) <0.01 or those that deviated from the expected Hardy-Weinberg equilibrium (*p* < 1.0 × 10^−4^) and SNPs with a call rate < 90% were removed. Then, 625,127 variants passed quality controls with a total genotyping rate of 0.9964. Finally, besides the GWAS analysis of all the chip’s SNPs that surpassed the quality controls, we undertook a search for SNPs of the *ADIPOQ* gene that were included in the OmniExpress-24 BeadChip genotyping array (v1.0 and v1.1) so as to specifically extract these results and analyze them as SNPs in candidate genes. Likewise, as the *CDH13* gene is another of the candidates that has been associated with adiponectin levels in many GWASs, we also pre-selected this gene as a candidate and extracted the SNPs that, for that gene, are found in the OmniExpress-24 BeadChip genotyping array. The corresponding genotype data were extracted by a Python script, created by the authors using the batch query resource SNP Report as previously reported [56]. Finally, the Python script produced the corresponding PLINK *.ped and *.map files of genotypes for statistical testing.

For SNP annotation (assigning the corresponding Gene Symbol to each rsID), we created a Phyton (version 3.7.4, 8 July 2019) script. This script uses an annotation database, designed and built with the database platform SQLite (version 3.33.0, 14 August 2020) (https://www.sqlite.org, accessed on 24 January 2021), containing data imported from the Infinium OmniExpress-24 BeadChip v1.2 genotyping array manifest (GRCh37) (https://emea.support.illumina.com/downloads/infinium-omniexpress-24-v1-2-product-files.html, accessed on 24 January 2021), and verified and completed with the NCBI SNP database (https://www.ncbi.nlm.nih.gov/snp/, accessed on 24 January 2021, GRCh37 and GRCh38) data. In the case of intergenic SNPs, no Gene Symbol is assigned.

### 2.5. Statistical Analysis

#### 2.5.1. General Associations and Candidate Gene Analysis

Plasma adiponectin and triglyceride levels were log-transformed (Ln) for the statistical analyses. First of all, we carried out descriptive statistical tests to summarize the characteristics of the sample studied. Chi-square tests were used to compare proportions. Student *t*-tests and ANOVA tests were applied to compare crude means of continuous variables. To estimate the association between adiponectin levels and plasma lipids, fasting glucose and adiposity-related variables, Spearman rank correlation coefficients were calculated. Analyses were carried out in the whole population and stratified by sex and diabetes status. To study the association between the four pre-selected SNPs (−11391 G/A, rs17300539; +45T > G, rs2241766; +276G > T, rs1501299; and rs17366568 G > A) in the *ADIPOQ* gene and plasma adiponectin levels, general linear models, unadjusted and adjusted for potential confounders, were used. Models were sequentially fitted as follows: model 1, unadjusted; model 2, adjusted for age and sex; model 3, additionally adjusted for type 2 diabetes; and model 4, additionally adjusted for BMI. Further adjustment for smoking, physical activity and adherence to the Mediterranean diet was carried out when indicated. Adjusted means for continuous variables were estimated from the multivariable adjusted general linear models. The genetic effect for SNPs was considered as additive (alleles 0, 1, and 2 for the minor allele). To test gene–diet interactions between the four pre-selected *ADIPOQ* polymorphisms and the Mediterranean diet pattern, we used the 14-item score as a categorical variable. To do so, we considered two categories based on the population mean: low adherence to the Mediterranean diet (8 points or lower) or high adherence to the Mediterranean diet (score of 9 points or higher). We also tested the interaction with the adherence variable as continuous with 14 points in order to compare results, when indicated. To analyze the gene-diet interactions in determining adiponectin concentrations, general linear hierarchical models were used in which the main effects and the interaction term were included. In each analysis, the confounding variables for adjustment were indicated in the tests. Besides testing the associations of the pre-selected polymorphisms in the *ADIPOQ* gene with adiponectin levels, associations with plasma lipid levels, fasting glucose and anthropometric variables were also tested for the most relevant pre-selected SNP. Likewise, gene-diet interactions with the Mediterranean diet for these variables were computed. In addition, the associations of the most significant *ADIPOQ* polymorphism, as well as the gene-diet interactions, considering prevalent type 2 diabetes as the outcome variable, were tested. Thus, uni- and multi-variant logistic regression models were used (including interaction terms or not, depending on the case). Odds ratios (OR) and 95% confidence intervals (CI) for the corresponding variables were estimated. Analyses were undertaken for the whole population and stratified by sex when indicated. These statistical analyses were performed with IBM SPSS Statistics (version 26.0), New York, NY, USA. All tests were two-tailed and *p*-values < 0.05 were considered statistically significant for these associations.

#### 2.5.2. GWASs Analyses

For the GWAS on adiponectin levels (Ln-transformed), association analyses were carried out using PLINK v1.9 [62,63]. Additive genetic models were fitted. General linear regression models were used, and regression coefficients and standard error (SE) were estimated. Model 1 (unadjusted), model 2 (adjusted for sex and age), model 3 (additionally adjusted for diabetes) and model 4 (adjusted for sex, age, diabetes and BMI) were computed. Beta regression coefficients for the minor allele were obtained. These analyses were performed in the whole population and stratified by sex and type 2 diabetes when indicated. Moreover, gene-Mediterranean diet interactions at the genome-wide level (including all the array SNPs that passed the quality control, as previously mentioned) in determining plasma adiponectin levels were analyzed. Additive genetic models were considered for the SNPs, and the Mediterranean diet was analyzed as categorical (low and high adherence). Hierarchical general linear regression models were fitted and the *p*-values for the SNP-diet interactions terms were computed.

We applied the conventional threshold of *p* < 5 × 10^−8^ for genome-wide statistical significance, as well as the standard *p*-value for suggestive genome-wide significance (*p* < 1 × 10^−5^).

We used Haploview (version 4.2) [64] and Functional Mapping and Annotation of Genome-Wide Association Studies (FUMA) [65] to create Manhattan plots and to calculate the linkage disequilibrium (LD) between the SNPs of interest. Quantile-quantile plots comparing the expected and observed *p*-values were performed in the R-statistical environment and with FUMA [65,66]. We used LocusZoom-Single Plot (http://locuszoom.org/; accessed on 24 January 2021) and LocusZoom.js (https://my.locuszoom.org/, accessed on 24 January 2021) to generate locus-specific graphical displays of the position of the selected SNPs in the GWAS to nearby genes and local recombination hotspots [67], as well as to indicate the LD. SNiPA, a tool for annotating and browsing genetic variants [68], was also used to display the LD between the selected SNPs and the nearby SNPs in regional plots using data from the European population based on the 1000 Genomes Project, incorporated in that tool.

Finally, we carried out additional association analyses for some top-ranked interesting SNPs as potentially novel signals for plasma adiponectin, including stratification by sex or by Mediterranean diet adherence strata, and additional adjustment for co-variates.

## 3. Results

### 3.1. Participant Characteristics

The demographic, anthropometric, clinical, biochemical and lifestyle characteristics of the study participants at baseline are presented in Table 1.

We included 954 subjects (348 men and 606 women) that, in addition to the general variables of the study, had plasma adiponectin measured at baseline available. They consisted of older men and women (mean age 67 ± 0.2 years). Mean adiponectin levels were 7.8 ± 0.2 for men and 11.9 ± 0.2 μg/mL for women. We used these values as sex-specific cut-off points to create a sex-specific dichotomous variable to define low and high adiponectin levels, taking into account the strong differences per sex. Prevalence of type 2 diabetes was high (47.2%) in the whole population. Obesity prevalence was also high (50.1% in the whole population).

### 3.2. Associations between Plasma Adiponectin and Fasting Glucose, Plasma Lipids, Adiposity Variables and Type 2 Diabetes

Table 2 shows associations between plasma adiponectin and fasting glucose, lipids and adiposity in the whole population and per sex. In the whole population, adiponectin was positively correlated with HDL-C (*r* = 0.39; *p* = 5.6 × 10^−36^) and negatively correlated with triglycerides (*r* = −0.28; *p* = 4.7 × 10^−18^) and fasting glucose (*r* = −0.26; *p* = 3.5 × 10^−16^). A significant inverse correlation at *p* = 0.006 was also found between adiponectin and waist circumference, although the correlation coefficient was relatively weak (*r* = −0.009). Similar results were found for men and women, although higher associations were detected in women.

Table 3 shows the association between adiponectin and type 2 diabetes at baseline. High plasma adiponectin was associated with lower type 2 diabetes risk in the whole population (OR = 0.61; 95%CI: 0.46–0.80; *p* = 4.4 × 10^−4^) for the dichotomous variable for adiponectin (high versus low concentrations), in a model adjusted for sex, age, BMI, smoking, physical activity and adherence to the Mediterranean diet. Likewise, when the continuous variable for adiponectin concentrations was used, we observed a strong inverse association, indicating the importance of the dose–effect. In the multivariate adjusted model, the OR was 0.47; 95%CI (0.35–0.63), *p* = 3.0 × 10^−7^, per unit increase in adiponectin. On analyzing the results per sex, higher protection was observed in women.

### 3.3. Association between the Pre-Selected ADIPOQ SNPs and Plasma Adiponectin Concentrations. Interactions with Adherence to the Mediterranean Diet

First, we analyzed the association between adiponectin and adherence to the Mediterranean diet score. We did not detect any statistically significant differences in plasma adiponectin depending on the low or high level of adherence to the Mediterranean diet (*p* = 0.531) after multivariate adjustment for sex, age, type 2 diabetes, BMI, smoking and physical activity. To identify specific foods relevant for adiponectin levels on this pattern, we also examined the association with specific food items included in the Mediterranean diet score. Sex-specific adiponectin levels were used, bearing in mind that adiponectin is significantly higher in women (*p* = 1.37 × 10^−30^). Thus, according to the cut-off values defined by the corresponding adiponectin means for men and women in Table 1, a dichotomous variable for sex-specific low and high adiponectin levels was created. In this categorical variable, low adiponectin levels were defined as having plasma adiponectin levels lower than 7.8 for men and less than 11.9 μg/mL for women. Likewise, high adiponectin levels were levels ≥ 7.8 or 11.9 μg/mL for men and women, respectively. Supplementary Table S2 shows the association between all the foods included in the Mediterranean diet adherence pattern score and the sex-specific adiponectin levels (low and high). No statistically significant associations for any food items were observed (*p* > 0.05 for all). Therefore, we used the total score for adherence to the Mediterranean diet as the main variable to test gene-diet interactions in further analyses.

Later, we examined the association between the four pre-selected candidate SNPs in the *ADIPOQ* gene: −11391 G/A (rs17300539); the T to G substitution in exon 2 (+45T > G, rs2241766); the G to T substitution in intron 2 (+276G > T, rs1501299); and rs17366568 (G > A) in intron 3, and adiponectin concentrations. Table 4 shows adiponectin means per genotype for each SNP. We only observed strong and additive associations for the −11391 G/A (rs17300539) polymorphism. The minor allele was associated with statistically significant higher adiponectin concentrations (*p* = 7.2 × 10^−5^) in the multivariable adjusted model.

For +276G > T (rs1501299), we obtained borderline significant associations. Interestingly, no statistically significant associations with adiponectin concentrations were observed for the rs17366568 (G > A) SNP (*p* = 0.257). This SNP was the most significantly associated with adiponectin levels (identified as the lead SNP) in the meta-analysis carried out in several European populations and published by Heid et al. (35). However, in this meta-analysis, the rs17366568 (G > A) SNP did not reach the statistical significance (*p* = 0.481) for the only Mediterranean population analyzed (from Italy, the InChianti study), suggesting potential population-specific differences in the *ADIPOQ* architecture and associations.

Regarding gene-diet interactions, we did not detect any statistically significant interactions between each one of the four pre-selected *ADIPOQ* candidate SNPs and adherence to the Mediterranean diet in determining plasma adiponectin concentrations in the whole population (*p* > 0.05 for all).

Supplemental Table S3 presents the associations and gene-diet interactions with the Mediterranean diet between the −11391 G/A (rs17300539) polymorphism and metabolic traits. Despite the strong association between this SNP and plasma adiponectin concentrations, no significant associations were obtained for this polymorphism and fasting glucose, BMI, waist circumference, HDL-C or type 2 diabetes.

### 3.4. GWAS for Plasma Adiponectin Concentrations in the Whole Population

We first carried out a GWAS for plasma adiponectin concentrations (ln-transformed) according to model 1 (unadjusted additive genetic model). We obtained several associations at the suggestive genome-wide significance level (*p* < 1.0 × 10^−5^). The lead SNP in this model was rs6705747, intergenic on chromosome 2 with *p* = 1.32 × 10^−7^ and MAF: 0.294. The second top-ranked SNP (*p* = 1.65 × 10^−6^) was rs11647294 (intronic) in the *VAT1L* (Vesicle Amine Transport 1 Like) gene on chromosome 16 with a high MAF (0.401) in comparison with the other top-ranked SNPs. This is the first time an SNP in the *VAT1L* gene is suggested to be related to adiponectin levels.

After adjustment for sex and age, the statistical significance for the *VAT1L* SNP was 6.9 × 10^−6^. After additional adjustment for diabetes and BMI, the statistical significance of the rs11647294-*VAT1L* SNP was attenuated (*p* = 1.0 × 10^−5^), and other SNPs reached more statistical significance. Supplemental Figure S1 presents the corresponding Manhattan plot showing the *p*-value (−log10 p) of each SNP analyzed in the model adjusted for sex, age, diabetes and BMI. Supplemental Figure S2 shows the corresponding Q-Q plot. Table 5 shows more detailed information on the top-ranked SNPs in the GWAS on plasma adiponectin concentrations adjusted for sex, age, type 2 diabetes and BMI, including the position, the effect (beta regression coefficient), the *p*-value, the corresponding MAF and the annotated gene.

**Figure 1:**
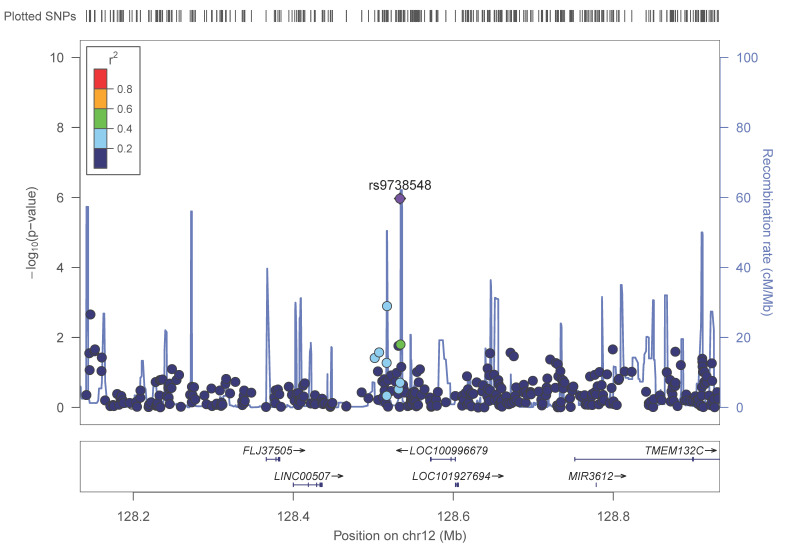
Regional plot for the lead SNP rs9738548, located on chromosome 12 (intergenic), in the GWAS for adiponectin concentrations in the whole population. *p*-values obtained in the general linear regression model adjusted for sex, age, diabetes and BMI.

After this adjustment for sex, age, type 2 diabetes and BMI, the lead SNP in the GWAS for adiponectin was rs9738548 (intergenic) on chromosome 12 (*p* = 1.1 × 10^−6^). Figure 1 shows the regional plot for this lead SNP. The MIR3612 gene is close to this intergenic SNP.

**Figure 2:**
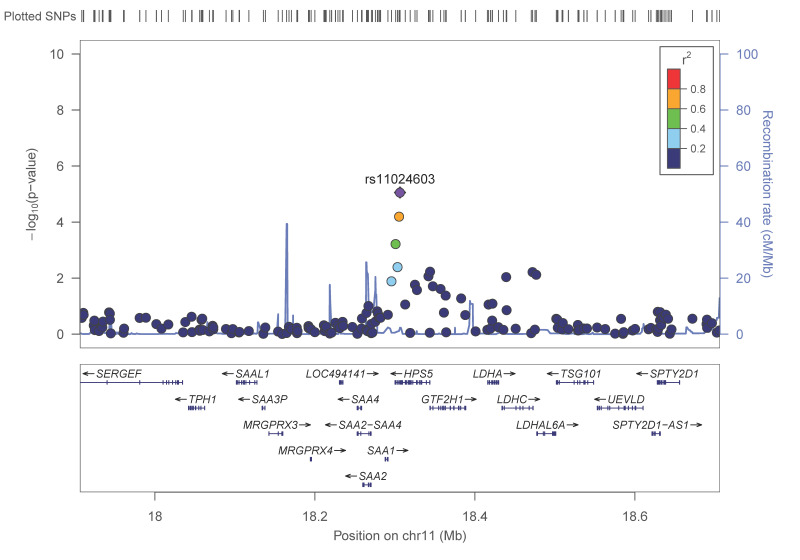
Regional plot for the SNP rs11024603, located in the Hermansky–Pudlak syndrome (HPS5) gene, on chromosome 11 in the GWAS, for adiponectin concentrations in the whole population. *p*-values obtained in the general linear regression model adjusted for sex, age, diabetes and BMI.

*RAB40C* (Ras-Related Protein Rab-40C) was also top-ranked at *p* = 4.4 × 10^−6^, but its MAF was relatively low. With a higher MAF (0.228), we detected an SNP in *HPS5* (Hermansky-Pudlak syndrome). Figure 2 depicts the regional plot for the rs1124603-*HPS5* SNP.

**Figure 3:**
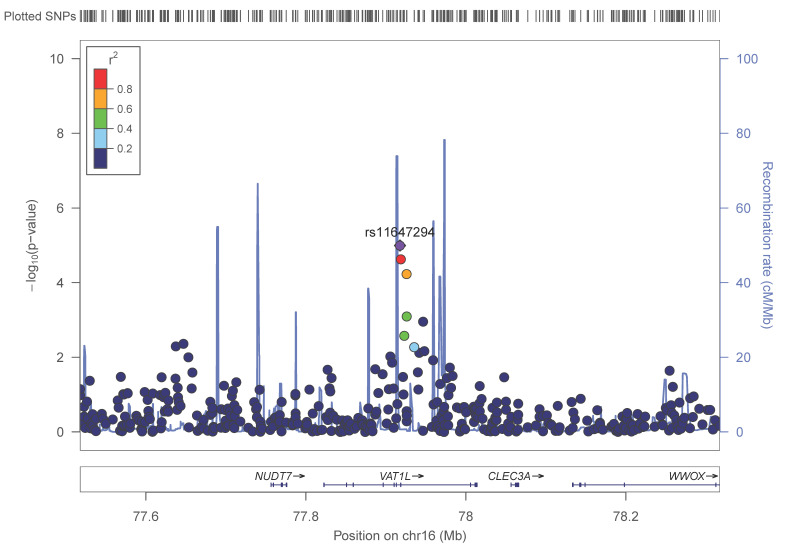
Regional plot for the SNP rs11647294, located in the vesicle amine transport 1 like (*VAT1L*) gene, on chromosome 11 in the GWAS, for adiponectin concentrations in the whole population. *p*-values obtained in the general linear regression model adjusted for sex, age, diabetes and BMI.

Figure 3 shows the regional plot corresponding to the rs11647294-*VATL1* SNP. This SNP is close to the *NUDT7* gene.

**Figure 4:**
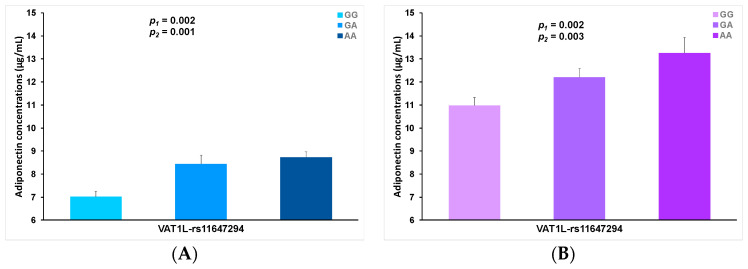
Plasma adiponectin concentrations (means) in men (**A**) and women (**B**) depending on the top-ranked rs11647294. *VATL1* SNP for the adiponectin GWAS. *p*-values (ln-transformed) were obtained in the general linear regression models: unadjusted (1) and adjusted for age, diabetes, BMI, smoking, physical activity and adherence to the Mediterranean diet (2). Error bars: (SE) of means. Genotype prevalence: GG (42.2%), GA (43.0%) and AA (14.8%).

We considered rs11647294-*VATL1* to be a promising candidate SNP for further analyses into plasma adiponectin concentrations and so studied the homogeneity of the association with adiponectin per sex strata. Figure 4 presents plasma adiponectin concentrations in men (panel A) and in women (panel B) depending on the rs11647294-*VATL1* SNP. This SNP was significantly associated with adiponectin concentrations, both in men and in women, in the unadjusted model. The associations remained statistically significant even after further adjustment for sex, age, diabetes, BMI, smoking, physical activity and adherence to the Mediterranean diet.

### 3.5. ADIPOQ and CDH13 Genes and Plasma Adiponectin Concentrations in the GWAS

As we did not observe a top-ranked signal for SNPs in the *ADIPOQ* and *CDH13* candidate genes, we examined the specific associations of these SNPs in the GWAS. We extracted all the *ADIPOQ* SNPs present in the OmniExpress Illumina array and checked the associations with plasma adiponectin concentrations. Supplemental Figure S3 presents the LD plot between the 10 *ADIPQ* SNPs included in the array and the pre-selected SNPs analyzed. The −11391 G/A (rs17300539) SNP was not included in the array, and its LD with other *ADIPOQ* SNPs is low. This SNP was detected at *p* = 7.2 × 10^−5^ in our previous candidate gene analysis with adiponectin concentrations (a little bit below the suggestive level of the GWAS significance, *p* = 1.0 × 10^−5^). Supplemental Table S4 shows the *p*-values, beta and MAF corresponding to the 10 *ADIPOQ* SNPs included in the array. The most significant *ADIPOQ* SNP in the model adjusted for sex, age, diabetes and BMI was the rs1501299 SNP, the only one that was common to both the array and our pre-selected list. The association with adiponectin was borderline significant, mimicking the results previously obtained in the candidate gene analysis.

Supplemental Table S5 presents the associations between the top-ranked *CDH13* SNPs of the *CDH13* SNPs included in the array and plasma adiponectin concentrations. Only one SNP reached statistical significance at the nominal *p*-value (rs4782726, *p* = 0.016), indicating that the *CDH13* gene is not a relevant locus for adiponectin concentrations in this Mediterranean population.

### 3.6. Stratified GWASs for Plasma Adiponectin

#### 3.6.1. Analysis per Sex

Considering the important differences in adiponectin concentrations between men and women, we conducted exploratory sex-specific GWASs. Supplemental Table S6 shows the top-ranked SNPs obtained in the sex-specific GWAS in men (A) and in women (B), after adjustment for age, BMI and type 2 diabetes. In men, we detected an interesting SNP, rs5876, located in MFSD14A (Hippocampus abundant transcript 1), also known as HIAT, on chromosome 1 (*p* = 2.0 × 10^−6^). Supplemental Figure S4 shows the LD regional plot for this SNP. For women, the most significant SNP was rs9989048 in the *SYT1* (Synaptotagmin-1) gene. Supplemental Figure S5 shows the LD regional plot for this SNP.

#### 3.6.2. Analysis per Diabetes Status

In the GWASs stratified by diabetes status (Table 6), we detected a statistically significant association at the GWAS level of significance in non-diabetic subjects for the rs2850066 SNP (*p* = 3.5 × 10^−8^), in the model adjusted for age, sex and BMI. This SNP is located in the *LOC101928269* gene on chromosome 21.

**Figure 5:**
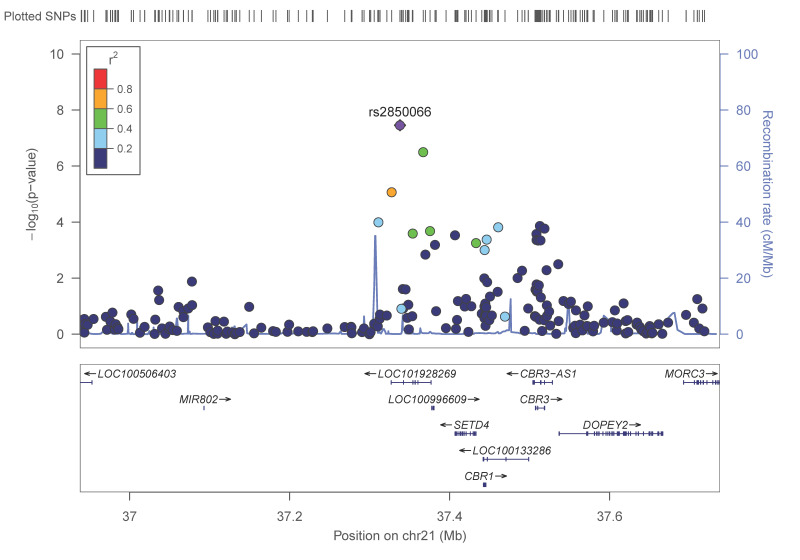
Regional plot for the SNP rs280066, located in the *LOC101928269* gene, on chromosome 21 in the GWAS, for adiponectin concentrations in non-diabetic subjects. *p*-values obtained in the general linear regression model adjusted for sex, age and BMI.

Figure 5 presents the regional plot for the rs2850066 SNP, located near the *MIR802* gene and near the *SETD4* (SET Domain Containing 4) genes.

Other SNPs in non-diabetic subjects reached the suggestive genome-wide level of significance (rs2835220, rs1283468, rs12887387, rs2146971 and rs11024603). In diabetic subjects, the most significant SNP was rs5992838 (intergenic) with *p* = 7.73 × 10^−6^. However, an interesting top-ranked SNP is rs7616406 in the *CAND2* (Cullin Associated And Neddylation Dissociated 2) gene on chromosome 3. Supplemental Figure S6 shows the regional plot for this SNP.

### 3.7. Gene–Mediterranean Diet Interactions for Adiponectin Concentrations at the Genome-Wide Level

Table 7 shows the top-ranked SNPs in the corresponding exploratory GWAS for the study of gene-Mediterranean diet interactions. Several SNPs reached the suggestive genome-wide level of significance for the gene-diet interaction term analyzed. The most significant interaction term with the Mediterranean diet adherence (low versus high) was for the rs17249128 SNP (*p* = 2.5 × 10^−7^), located in *LOC101927334* on chromosome 16. Supplemental Figure S7 shows the LD regional plot for this SNP.

Table 7 also shows the beta coefficients for each stratum. Beta 1 corresponds to the effect of the minor allele of the indicated SNP on plasma adiponectin concentrations in the low adherence to the Mediterranean diet, and beta 2 indicates the regression coefficient for the minor allele for high adherence to the Mediterranean diet. The MAF for the top-ranked SNP (rs17249128) is high (0.466), being an interesting candidate SNP for further investigation. Another very remarkable SNP in the top-ranked list of gene-diet interactions is rs2917570 in *OPCML* (Opioid Binding Protein/Cell Adhesion Molecule Like) on chromosome 3. Interestingly, SNPs in the *OPCML* gene have been associated with adiponectin concentrations in previous GWASs carried out in some European populations [35], but not in others [35]. The existence of a gene-diet interaction may explain these previous results. In our study, when adherence to the Mediterranean diet is low, the minor allele of the rs2917570 SNP is associated with higher adiponectin concentrations (B = 0.113, *p* = 0.008). However, when adherence to the Mediterranean diet is high, the minor allele is associated with lower adiponectin concentrations (B= −0.118, *p* = 0.003); *p* for this SNP–Mediterranean diet interaction is 5.1 × 10^−6^.

**Figure 6:**
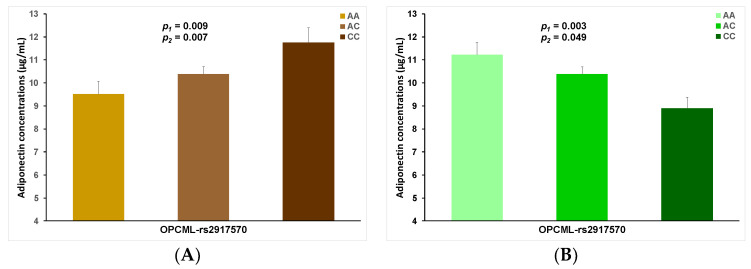
Gene–diet interaction effect between the SNP rs2917570, located in the opioid-binding protein/cell adhesion molecule (*OPCML*), chromosome 11, and adherence to the Mediterranean diet: low adherence (panel (**A**)) and high adherence (panel (**B**)) on plasma adiponectin concentrations (ln-transformed) in the whole population. *p*-values were obtained in the general linear regression models: unadjusted (1) and adjusted for sex, age, diabetes, BMI, smoking and physical activity (2). Error bars: (SE) of means. Genotype prevalence: AA (25%), AC (54%) and CC (21%).

Figure 6 depicts such a gene-Mediterranean diet interaction in panels A (low adherence) and B (high adherence). Additional adjustment for age, diabetes, BMI, smoking and physical activity did not change the statistical significance level for the associations.

Finally, we would like to mention that we obtained a top-ranked gene-Mediterranean diet interaction signal (*p* = 1.5 × 10^−5^) involving *PARP1* (Poly(ADP-Ribose) Polymerase 1). This gene has been related to adiponectin gene expression [69,70] and some dietary modulation has been reported in animal models [70,71].

## 4. Discussion

In this study carried out in a high-cardiovascular risk Mediterranean population, we found that plasma adiponectin levels were higher in women and directly associated with higher HDL-C and inversely associated with fasting triglycerides and fasting glucose levels, as well as with lower type 2 diabetes risk, even after multivariate adjustment. These findings are in accordance with many previous reports in other populations of various ethnic backgrounds [72,73,74,75,76]. The robust linear inverse association between plasma adiponectin concentrations and type 2 diabetes risk observed in our population, mainly in women, is concordant with other reports where associations with adiponectin levels were stronger in individuals with a higher metabolic risk profile [77,78,79], supporting the idea that the association between adiponectin concentrations and type 2 diabetes is stronger in subjects with higher BMI.

In our population, the direct and inverse associations between plasma adiponectin and fasting glucose, type 2 diabetes or lipid levels remained statistically significant even after adjustment for BMI. The mechanisms explaining these protective associations are far from being determined, but there is some agreement suggesting that the anti-inflammatory properties of adiponectin [15,80] are likely to be the major component of its beneficial effects against insulin resistance [15,16,17,76,81,82]. However, in this study, we did not have data on inflammation markers for most of the participants, so we were unable to analyze these associations. We did, nevertheless, have data on genetic polymorphisms, both at the candidate gene level and at the genome-wide level, and found some interesting suggestive associations. Firstly, we set out to analyze the associations between four well-known candidate SNPs in the *ADIPOQ* gene and plasma adiponectin levels, subsequently carrying out an exploratory GWAS, given that very few reports have focused on Mediterranean subjects (mainly those including Italian participants) and it is known that even though specific GWAS-population associations may exist, they can misestimate across global populations [83,84]. Although many GWASs have been carried out on adiponectin in populations of Europe or with European ancestry in North America [35,41,42,43,44,45], the Spanish Mediterranean population has not been included in these meta-analyses. Thus, it is necessary to carry out specific studies on this population.

Despite the small sample size of our study (954 participants), we opted to undertake an exploratory GWAS to at least identify the genetic variants most associated with plasma adiponectin levels in high-cardiovascular risk Mediterranean subjects. In previous studies on Spanish Mediterranean individuals, we were able to detect, at the GWAS level of significance, the main expected top-ranked SNPs in genes consistently associated with several traits with a strong genetic influence (plasma bilirubin, polyunsaturated fatty acids and bitter taste perception), with even smaller sample sizes [85,86,87]. For adiponectin, if the genetic association between the main SNPs in candidate genes consistently reported in other populations (*ADIPOQ* or *CDH13*) is strong, our sample size would be enough to detect them at the GWAS level. Thus, in a previous GWAS carried out on a Korean population [34], with an initial sample size similar to ours (about 950 participants), the authors detected some statistically significant associations at the GWAS level with SNPs in the *CDH13* gene, which is more important in Asian populations than in European ones [34,35,36,37,38,39,40,41,42,43,44,45]. In this Korean population [34], the top-ranked SNP was rs3865188-*CDH13* with MAF: 0.309, reaching a *p*-value of 5 × 10^−15^. In our study, we did not detect any association at the GWAS level (*p* < 5 × 10^−8^) or at the suggestive level of significance (*p* < 1 × 10^−5^) with SNPs in the *CDH13* gene and adiponectin concentrations.

In our exploratory GWAS, for adiponectin concentrations in the whole population, we detected some signals at the suggestive level of GWAS significance (*p* < 1 × 10^−5^), including the rs9738548 (intergenic) SNP on chromosome 12, the rs11647294-*VAT1L* SNP on chromosome 16, the rs12149200-RAB40C SNP on chromosome 16 and the rs1124603-HPS5 SNP on chromosome 11, among others. As far as we know, this is the first time that our top-ranked SNPs have been reported as being associated with adiponectin concentrations. Some of these signals may be false positive associations, and replication, mainly in this population and in other populations, is needed to confirm the signals. Several SNPs on chromosome 12 have been identified as genome-wide signals in the adiponectin meta-analysis of 45,891 individuals carried out by Dastani et al. [41]. These signals were the following: rs601339-GPR109A; rs6488898-ATP6V0A2; rs7133378-DNAH10; rs7305864-CCDC92; and rs7978610-ZNF664. Likewise, in this meta-analysis [41], several SNPs on chromosome 16 reached the genome-wide level of significance. In addition to the strong signals on CDH13, located on chromosome 16, other genome-wide signals included the rs2925979-CMIP SNP. Our top-ranked SNPs also included two SNPs on chromosome 16 (rs11647294-*VAT1L* and rs12149200-RAB40C). Among these top-ranked SNPs for plasma adiponectin in this high-cardiovascular risk Mediterranean population, we suggest that an interesting candidate would be the rs11647294-*VAT1L* SNP. This SNP has a high MAF (0.401), supporting a more stable association with adiponectin concentrations in comparison with the potential bias related to low-frequency variants in studies with a small sample size. Thus, it has been reported that common SNPs (MAF: 0.25–0.50) result in significantly fewer false positives than for less common SNPs [88]. Moreover, we conducted a stratified analysis per sex between the rs11647294-*VAT1L* SNP and adiponectin concentrations in men and women, and we observed statistically significant associations in both men and women strata, therefore increasing the level of evidence against a false positive association. The *VAT1L* gene has been associated with schizophrenia in some GWASs and subsequent pathway enrichment analysis suggested its involvement in neural and immune system-related pathways [89]. Likewise, the *VAT1L* gene has been associated with the von Economo neurons in brain functional analyses [90] and in calcific aortic valve disease [91], among other diseases [92,93]. Functionally, the *VAT1L* gene has been linked to several immune system pathways [89,91], and adiponectin levels could be related to these pathways [15,94].

Regarding the *ADIPOQ* SNPs, we did not identify such SNPs among the top-ranked in any of the GWAS strata of this population, as initially expected according to the GWASs carried out in several European populations [41,42,43,44,45]. We, therefore, proceeded to extract all the *ADIPOQ* SNPs that were in the array to carry out a more detailed study. For the *ADIPOQ* gene, the number of SNPs included in the array was low (*n* = 10) and none of them showed statistical significance at the *p* < 0.05 level. The most significant, although without reaching *p* < 0.05, was rs1501299, which had already been previously analyzed in our initial list of the four pre-selected candidate SNPs in the *ADIPOQ* gene, therefore being consistent with our previous findings. The association between the candidate SNPs +276G > T (rs1501299) and +45T > G (rs2241766) with plasma adiponectin has been heterogeneous across diverse populations [29,41,46,47,48,49,50,95,96]. Of the four candidate SNPs analyzed, we only observed strong and additive associations for the −11391 G/A (rs17300539) polymorphism, in agreement with several studies showing significant associations for this variant [49,50]. However, we did not find any significant association for the rs17366568 (G > A) SNP, which was the SNP most significantly associated with adiponectin levels in the meta-analysis carried out by Heid et al. in European populations [35]. Interestingly, in this meta-analysis, despite the global association, when we look at specific populations, in agreement with our results, the rs17366568 SNP did not reach statistical significance for the only Mediterranean population analyzed (Italian subjects from the InChianti study, Italy) [35], therefore increasing the evidence of potential population-specific differences.

On the other hand, the −11391 G/A (rs17300539) polymorphism in the *ADIPOQ* gene was significantly associated (*p* = 7.2 × 10^−5^) with adiponectin concentrations in our Mediterranean population in the separate candidate gene analysis. However, we did not observe such association in the GWAS because this SNP was not included in the array. If we had carried out an imputation analysis, we could have possibly detected this signal or that of other SNPs in the *ADIPOQ* gene among the top-ranked, but without reaching the GWAS level (because this association, despite being high, did not reach the GWAS level of statistical significance, nor the suggestive significance level, although being close). In our case, we preferred to undertake the exploratory GWAS with directly analyzed SNPs without using imputations in order to avoid the bias due to low imputation quality due to a not very large sample size.

Furthermore, we carried out sex-specific and diabetes-specific GWASs to detect if the high-cardiovascular risk characteristics of our population provide a profile of associations in candidate genes that differs from the most commonly studied populations in other GWASs, from which diabetic subjects are sometimes excluded and which generally include a younger population with a lower cardiovascular risk. Having consulted the published GWAS results in greater detail [34,35,36,37,38,39,40,41,42,43,44,45], we observed that although the greatest associations are found for SNPs in the *ADIPOQ* and *CDH13* genes, the other top-ranked genes differ widely between populations, indicating great heterogeneity in the findings depending on the participant characteristics. This fact deserves much more attention, since the SNPs in the two top-ranked genes explain a very low percentage of adiponectin variance and there will surely be many other genes associated with it, but whose contribution may be highly variable and dynamic depending on the geographic origin, age, sex and the presence or absence of obesity and/or diabetes in the analyzed populations. Therefore, subsequent studies should take this perspective into account when interpreting the results and undertaking statistical analyses by presenting more information on the top-ranked SNPs in each population analyzed, instead of presenting the summary of GWAS meta-analyses.

One limitation of our study is the small sample size compared with other GWASs undertaken on other populations. This limits its statistical power, as clearly it would have been much better to have studied a larger population. However, as the Spanish Mediterranean population is under-represented in the studies published so far, it is of great interest to have these results available, given that they are the first on this population.

Moreover, despite our small sample size, in our exploratory gene–diet interaction study, we detected some suggestive gene-Mediterranean diet interactions on analyzing SNPs at the genome-wide level. Among them, we consider as promising for further analysis the interaction found with the rs2917570 SNP in the *OPCML* gene. SNPs in this gene have been associated with adiponectin concentrations in previous GWASs in some European populations [35], but replication is low. The existence of a gene–diet interaction may help to explain some previous inconsistent results. Likewise, another relevant gene-Mediterranean diet interaction to be explored in further studies is that involving the *PARP1* gene. This gene has been related to adiponectin gene expression in functional studies [69,70] and some dietary modulation has been reported in adipose tissue of mice in response to high-fat diet feeding or calorie restriction [71]. These previous results contribute to increase the importance of the observed gene-Mediterranean diet interaction encompassing the rs3219110-*PARP1* polymorphism. As this work is only statistical and exploratory, we could not undertake a mechanistic study to suggest potential mechanisms explaining these SNP–diet interactions. With our results, other authors will be able to carry out additional studies to better characterize the possible interaction mechanisms.

## 5. Conclusions

Our results indicate that plasma adiponectin is strongly associated with a favorable lipid profile (high HDL-C and low triglycerides) and inversely related to type 2 diabetes risk in this high-cardiovascular risk Mediterranean population, in agreement with previous results in diverse populations. However, the genetic variants more associated with plasma adiponectin concentrations could be population-specific and influenced by sex and diabetes status. Moreover, in our exploratory analysis, despite the small sample size, we detected some suggestive gene-Mediterranean diet interactions at the GWAS level that may be interesting for further characterization in later studies in this population and in other populations.

## Figures and Tables

**Table 1:** Demographic, clinical biochemical and lifestyle characteristics of the study population.

	Total (*n* = 954)	Men (*n* = 348)	Women (*n* = 606)	*p*
Age (years)	67.0 ± 0.2	66.2 ± 0.4	67.4 ± 0.2	0.007
Weight (kg)	76.3 ± 0.4	81.5 ± 0.6	73.4 ± 0.4	<0.001
BMI (kg/m^2^)	30.3 ± 0.1	29.6 ± 0.2	30.7 ± 0.2	<0.001
Waist circumference (cm)	102.5 ± 0.4	104.2 ± 0.6	101.5 ± 0.5	0.001
SBP (mm Hg)	146.8 ± 0.7	148.3 ± 1.1	145.9 ± 0.9	0.086
DBP (mm Hg)	81.9 ± 0.3	82.6 ± 0.6	81.4 ± 0.4	0.099
Total cholesterol (mg/dL)	207.7 ± 1.3	199.7 ± 2.0	212.3 ± 1.6	<0.001
LDL-C (mg/dL)	129.1 ± 1.2	124.5 ± 1.9	131.6 ± 1.5	0.004
HDL-C (mg/dL)	52.5 ± 0.4	47.9 ± 0.6	55.1 ± 0.6	<0.001
Triglycerides ^1^ (mg/dL)	131.5 ± 2.3	136.6 ± 3.7	128.6 ± 2.8	0.051
Fasting glucose (mg/dL)	120.4 ± 1.3	128.3 ± 2.2	115.8 ± 1.5	<0.001
Plasma adiponectin ^2^ (µg/mL)	10.4 ± 0.2	7.8 ± 0.2	11.9 ± 0.2	<0.001
Physical activity (MET-min/day)	166 ± 6	216 ± 12	137 ± 5	<0.001
Adherence to MedDiet (P14) ^3^	8.0 ± 2.8	7.9 ± 2.8	8.1 ± 2.7	0.130
High adherence MedDiet ^4^	476 (50.0)	177 (51.0)	299 (49.4)	0.637
Current smokers: *n*, %	116 (12.2)	88 (25.3)	28 (4.6)	<0.001
Type 2 diabetes: *n*, %	450 (47.2)	197 (56.6)	253 (41.7)	<0.001
Obesity: *n*, %	478 (50.1)	151 (43.4)	327 (54.0)	0.002

Values are mean ± standard error (SE) for continuous variables and number (%) for categorical variables. BMI: body mass index; SBP: systolic blood pressure; DBP: diastolic blood pressure; LDL-C: high-density lipoprotein cholesterol; HDL-C: low-density lipoprotein cholesterol; MET: metabolic equivalent.; P: *p*-value for the comparisons between men and women. Student’s *t* tests were used to compare means and chi-squared tests were used to compare categories. ^1^: Triglycerides were ln-transformed for statistical testing. ^2^: Adiponectin concentrations were ln-transformed for statistical testing. ^3^: Using the 14-item questionnaire for adherence to the Mediterranean diet (MedDiet). ^4^: High adherence to the MedDiet: score ≥ 9.

**Table 2:** Association between plasma adiponectin levels and metabolic traits in the whole population and per sex.

Trait ^1^		Total Adiponectin (µg/mL)	Men Adiponectin (µg/mL)	Women Adiponectin (µg/mL)
Fasting glucose (mg/dL)	*r* ^2^	−0.261	−0.109	−0.271
	*p* ^3^	*3.48 × 10^−16^*	*0.043*	*1.42 × 10^−11^*
Tryglicerides (mg/dL)	*r* ^2^	−0.276	−0.169	−0.340
	*p* ^3^	*4.72 × 10^−18^*	*1.59 × 10^−3^*	*8.17 × 10^−18^*
HDL-C (mg/dL)	*r* ^2^	0.391	0.292	0.342
	*p* ^3^	*5.59 × 10^−36^*	*3.03 × 10^−8^*	*5.00 × 10^−18^*
LDL-C (mg/dL)	*r* ^2^	0.109	0.084	0.077
	*p* ^3^	*8.52 × 10^−4^*	*0.122*	*0.059*
BMI (Kg/m^2^)	*r* ^2^	0.029	0.068	−0.075
	*p* ^3^	*0.367*	*0.209*	*0.064*
Waist circumference (cm)	*r* ^2^	−0.090	0.032	−0.093
	*p* ^3^	*0.006*	*0.559*	*0.024*

^1^: Metabolic traits measured in fasting status for the whole population (*n* = 954 participants, including *n* = 348 men and *n* = 606 women). ^2^: *r* (normal) is the Spearman correlation coefficient. ^3^: *p*-value (italics) for the Spearman correlation coefficient.

**Table 3:** Association between adiponectin levels (as dichotomous and continuous variable) and type 2 diabetes in the whole population and in men and women.

	Total		Men		Women	
Adiponectin	OR ^3^ and 95% CI	*p* ^5^	OR ^3^ and 95% CI	*p* ^5^	OR ^3^ and 95% CI	*p* ^5^
Dichotomous variable ^1^						
Model 1 ^2^	0.65 (0.49–0.84)	0.001	0.77 (0.50–1.19)	0.239	0.59 (0.42–0.82)	0.002
Model 2 ^3^	0.61 (0.46–0.79)	2.80 × 10^−4^	0.68 (0.43–1.06)	0.090	0.57 (0.41–0.81)	0.001
Model 3 ^4^	0.61 (0.46–0.80)	4.40 × 10^−4^	0.67 (0.42–1.07)	0.093	0.59 (0.42–0.85)	0.004
Continuous variable ^6^						
Model 1 ^2^	0.46 (0.35–0.59)	3.00 × 10^−9^	0.69 (0.48–1.09)	0.097	0.42 (0.29–0.59)	1.01 × 10^−6^
Model 2 ^3^	0.47 (0.35–0.62)	1.41 × 10^−7^	0.64 (0.39–1.03)	0.065	0.40 (0.28–0.57)	6.40 × 10^−7^
Model 3 ^4^	0.47 (0.35–0.63)	3.03 × 10^−7^	0.64 (0.39–1.03)	0.066	0.42 (0.29–0.61)	3.10 × 10^−5^

OR: odds ratio; CI: confidence interval. ^1^: A categorical variable for low and high adiponectin concentration was created taking into account sex-specific levels (high ≥7.8 (µg/mL) for men, and ≥11.9 (µg/mL) for women). OR indicates the risk for high level versus low level. ^2^: Unadjusted regression model. ^3^: Model adjusted for sex, age and BMI. ^4^: Model adjusted for sex, age, BMI, physical activity, current smokers and adherence to the Mediterranean diet. ^5^: *p*-value obtained in the corresponding logistic regression model. ^6^: Adiponectin (µg/mL) was used as a continuous variable (ln-transformed) and the OR indicates the risk per unit.

**Table 4:** Association between the 4 pre-selected *ADIPOQ* candidate single-nucleotide polymorphisms (SNPs) and adiponectin concentrations in the whole population and interaction with adherence to the Mediterranean diet.

Pre-Selected *ADIPOQ* Candidate SNPs	Genotypes					
	11 Mean ± SE	12 Mean ± SE	22 Mean ± SE	*p* ^1^	*p* ^2^	*p-Int*^3^ MedDiet
−11391 G > A (rs17300539) ^4^	10.09 ± 0.20	11.45 ± 0.47	13.45 ± 1.00	4.80 × 10^−4^	7.20 × 10^−5^	0.559
+45T > G (rs2241766) in exon 2 ^5^	10.41 ± 0.22	10.37 ± 0.36	11.40 ± 0.89	0.436	0.808	0.072
+276G > T (rs1501299) in intron 2 ^6^	10.33 ± 0.25	10.26 ± 0.30	12.02 ± 0.69	0.091	0.047	0.795
rs17366568 (G > A) ^7^	10.49 ± 0.20	10.39 ± 0.46	8.57 ± 1.39	0.271	0.257	0.525

Multivariable adjusted models and gene-diet interactions with adherence to the Mediterranean diet. 11, 12 and 22 indicate the three genotypes for each SNP: 1 major allele, 2 minor alleles. Mean adiponectin concentrations are expressed in µg/mL. ^1^: *p*-value obtained in the unadjusted general linear additive model. ^2^: *p*-value obtained in the general linear additive model adjusted for sex, age, diabetes, BMI, physical activity, smoking and adherence to the Mediterranean diet. ^3^: *p*-value for the interaction term between the corresponding SNP and adherence to the Mediterranean diet in the multivariable model 2. ^4^: Genotype prevalence was 78.4% GG; 19.6% GA; and 2.0% AA (*n* = 951). ^5^: Genotype prevalence was 67.2% TT; 28.9% TG; and 3.9% GG (*n* = 954). ^6^: Genotype prevalence was 51.9% GG; 39.2% GT; and 8.9% TT (*n* = 941). ^7^: Genotype prevalence was 79.4% GG; 19.3% GA; and 1.3% AA (*n* = 948). Ln-transformed adiponectin (µg/mL) for *p*-values.

**Table 5:** Top-ranked SNPs in the genome-wide association study (GWAS) for adiponectin concentrations (ln-transformed) in the whole population.

Chr	SNP	BP	Beta	*p*	Alleles	MAF	Strand	Gene
12	rs9738548	128533430	0.141	1.07 × 10^−6^	T	0.137	+	intergenic
13	rs396318	56590135	0.247	3.03 × 10^−6^	G	0.129	+	intergenic
4	rs17613848	23485012	−0.123	3.72 × 10^−6^	A	0.135	+	*LOC105374524*
16	rs12149200	678430	0.184	4.43 × 10^−6^	T	0.020	+	*RAB40C*
4	rs998888	160494830	0.215	8.02 × 10^−6^	T	0.097	-	*LOC107986324*
11	rs11024603	18306399	0.129	8.77 × 10^−6^	A	0.228	+	*HPS5*
1	rs6698721	185849364	0.446	1.00 × 10^−5^	A	0.176	+	*HMCN1*
16	rs11647294	77917661	0.098	1.01 × 10^−5^	T	0.401	+	*VAT1L*
21	rs2850066	37338018	−0.119	1.02 × 10^−5^	G	0.273	+	*LOC101928269*
16	rs4785550	48864323	0.183	1.05 × 10^−5^	T	0.227	+	intergenic

Model adjusted for sex, age, diabetes and BMI. BMI: body mass index. Chr: chromosome. SNP: single-nucleotide polymorphism. BP: base position in the chromosome (Homo Sapiens GRCh37.p13 genome build used in Illumina HumanOmniExpress-24 BeadChip). Beta: indicates the effect for the minor allele on adiponectin (ln-transformed). P: *p*-value obtained in the multivariable linear regression model adjusted for sex, age, diabetes and BMI for each SNP using a genetic additive model. MAF: minor allele frequency.

**Table 6:** Top-ranked SNPs of the GWAS for adiponectin concentrations (ln-transformed) in non-diabetic and diabetic subjects.

**A: Non-Diabetic Subjects**								
**Chr**	**SNP**	**BP**	**Beta**	***p***	**Alleles**	**MAF**	**Strand**	**Gene**
21	rs2850066	37338018	−0.189	3.51 × 10^−8^	G	0.273	+	*LOC101928269*
21	rs2835220	37367098	−0.157	3.18 × 10^−7^	C	0.283	+	*LOC101928269*
6	rs1283468	70038147	0.200	4.52 × 10^−7^	A	0.180	+	*ADGRB3*
14	rs12887387	90196183	0.232	1.71 × 10^−6^	T	0.162	+	intergenic
14	rs2146971	90173453	0.198	2.11 × 10^−6^	T	0.161	-	intergenic
11	rs11024603	18306399	0.184	2.36 × 10^−6^	A	0.228	+	*HPS5*
**B: Diabetic Subjects**								
**Chr**	**SNP**	**BP**	**Beta**	***p***	**Alleles**	**MAF**	**Strand**	**Gene**
22	rs5992838	18264831	−0.147	7.73 × 10^−6^	G	0.411	+	intergenic
10	rs11239763	43262368	−0.161	1.07 × 10^−5^	C	0.393	+	*LOC105378269*
9	rs1779307	91449366	0.144	1.09 × 10^−5^	C	0.356	+	intergenic
10	rs11239766	43264591	−0.161	1.35 × 10^−5^	G	0.386	+	*LOC105378269*
2	rs2290130	232263127	0.168	1.35 × 10^−5^	A	0.233	-	*B3GNT7*
3	rs7616406	12862257	0.152	1.49 × 10^−5^	G	0.446	+	*CAND2*

Model adjusted for sex, age and BMI. BMI: body mass index. Chr: chromosome. SNP: single-nucleotide polymorphism. BP: base position in the chromosome (Homo Sapiens GRCh37.p13 genome build used in Illumina HumanOmniExpress-24 BeadChip). Beta: indicates the effect for the minor allele on adiponectin concentrations (ln-transformed). P: *p*-value obtained in the multivariable linear regression model adjusted for sex, age and BMI for each SNP using a genetic additive model. MAF: minor allele frequency.

**Table 7:** Top-ranked SNPs of the GWAS for gene-diet interactions between the genome-wide SNPs and adherence to the Mediterranean diet in determining adiponectin concentrations (ln-transformed) in the whole population.

Chr	SNP	Beta 1	Beta 2	*p*-Interaction ^1^	Alleles	MAF	Strand	Gene
16	rs17249128	−0.125	0.123	2.53 × 10^−7^	G	0.466	+	*LOC101927334*
4	rs828154	0.077	−0.191	9.74 × 10^−7^	G	0.485	−	intergenic
3	rs326251	0.131	−0.097	4.20 × 10^−6^	C	0.333	−	intergenic
11	rs2917570	0.113	−0.118	5.12 × 10^−6^	T	0.497	+	*OPCML*
4	rs13111850	0.334	−0.130	6.42 × 10^−6^	C	0.033	+	intergenic
2	rs6433691	0.197	−0.162	6.45 × 10^−6^	A	0.241	+	*PDE11A*
4	rs10019416	−0.093	0.175	7.46 × 10^−6^	A	0.258	+	*SLIT2*
11	rs3016384	0.118	−0.109	8.05 × 10^−6^	T	0.491	−	*OPCML*
2	rs3770019	0.206	−0.156	1.09 × 10^−5^	C	0.141	+	*PDE11A*
8	rs13280216	0.174	−0.100	1.16 × 10^−5^	A	0.267	+	intergenic
2	rs9677333	0.203	−0.156	1.31 × 10^−5^	C	0.139	+	*PDE11A*
4	rs10023405	−0.119	0.112	1.39 × 10^−5^	G	0.210	+	*KIAA1211*
10	rs912745	−0.148	0.211	1.44 × 10^−5^	T	0.092	+	*LOC102724627*
1	rs3219110	0.061	−0.146	1.51 × 10^−5^	C	0.319	−	*PARP1*

Chr: chromosome. SNP: single-nucleotide polymorphism. BP: base position in the chromosome (Homo Sapiens GRCh37.p13 genome build used in Illumina HumanOmniExpress-24 BeadChip). Beta: indicates the effect for the minor allele on adiponectin concentrations (ln-transformed). Beta 1: indicates the regression coefficients for the low adherence to the Mediterranean diet strata (50%). Beta 2: indicates the regression coefficients for the high adherence to the Mediterranean diet strata, based on the population mean (9 points). ^1^: *p-*value obtained for the interaction term SNP-adherence to the Mediterranean diet in the corresponding hierarchical general linear regression model including the main effects and interaction terms in the whole population. MAF: minor allele frequency.

## Data Availability

The data are not publicly available due to informed consent restrictions. Potential collaborations will be available on request from the corresponding author.

## Supplementary Materials

The following are available online at https://www.mdpi.com/2072-6643/13/2/541/s1, Table S1: Quantitative 14-item questionnaire for adherence to the Mediterranean diet.; Table S2: Association between plasma adiponectin (low versus high levels using sex-specific cut-off) and the 14-item Mediterranean diet score in the whole population.; Table S3: Association between the rs17300539-*ADIPOQ* promoter polymorphism and metabolic traits; Table S4: SNPs of the *ADIPOQ* gene (included in the Illumina array) for adiponectin concentrations (ln-transformed) in the whole population.; Table S5: Top-ranked SNPs of the *CDH13* gene for adiponectin concentrations (ln-transformed) in the whole population.; Table S6: Top-ranked SNPs of the GWAS for adiponectin concentrations (ln-transformed) in men and women; Figure S1: Manhattan plot for the SNP-based GWAS on adiponectin concentrations (ln-transformed) in the whole population.; Figure S2: Q-Q plot for the SNP-based GWAS on adiponectin concentrations (ln-transformed) in the whole population.; Figure S3: Linkage disequilibrium (LD) r2 plot for the SNPs of the *ADIPOQ* gene (included in the Illumina array and pre-selected), on chromosome 3.; Figure S4: Linkage disequilibrium (LD) regional plot for the SNP rs5876, located in the hippocampus abundant transcript 1 (HIAT) gene, also known as the MFSD14A gene, on chromosome 1 in the GWAS, for adiponectin concentrations in men; Figure S5: Linkage disequilibrium (LD) regional plot for the sentinel SNP rs9989048-Synaptotagmin 1 (*SYT1*), on chromosome 12, and the regional plot obtained in the GWAS for adiponectin concentrations (ln-transformed) in women.; Figure S6: Regional plot for the sentinel SNP rs7616406-*CAND2*, on chromosome 3.; Figure S7: Linkage disequilibrium (LD) plot for the lead (sentinel) SNP rs17249128-*LOC101927334*, on chromosome 16, in the GWAS for the gene-Mediterranean diet interactions on plasma adiponectin concentrations (ln-transformed).
