# Supplementary material for: Circulating Adiponectin and Its Association with Metabolic Traits and Type 2 Diabetes: Gene-Diet Interactions Focusing on Selected Gene Variants and at the Genome-Wide Level in High-Cardiovascular Risk Mediterranean Subjects
Source: Nutrients. 2021 Feb 7;13(2):541. doi: 10.3390/nu13020541 (PMC7914877; doi:10.3390/nu13020541)

ONLINE SUPPORTING MATERIAL

Circulating adiponectin and its association with metabolic traits and type 2 diabetes: gene-diet interactions focusing on selected gene variants and at the genome-wide level in high cardiovascular risk Mediterranean subjects

Oscar Coltell, Carolina Ortega-Azorín, Jose V. Sorlí, Olga Portolés, Eva M. Asensio, Carmen Saiz, Rocío Barragán, Ramon Estruch and Dolores Corella

Supplemental Table 1. 2
Supplemental Table 2 3
Supplemental Table 3 4
Supplemental Figure 1 4
Supplemental Figure 2 5
Supplemental Figure 3 5
Supplemental Table 4 6
Supplemental Table 5 7
Supplemental Table 6 8
Supplemental Figure 4 9
Supplemental Figure 5 9
Supplemental Figure 6 10
Supplemental Figure 7 10

Supplemental Table 1. Quantitative 14-item questionnaire for Adherence to Mediterranean diet.

Food items and frequency of consumption		Criteria for 1 point[1]
1.	Do you use olive oil as main culinary fat?	Yes
2.	How much olive oil (tablespoons) do you consume in a given day (including oil used for frying, salads, out of house meals, etc.)?	≥4
3.	How many vegetable servings do you consume per day? (1 serving = 200g [consider side dish as half serving])	≥2 (≥1 portion raw or as salad)
4.	How many fruit units (including natural fruit juices) do you consume per day?	≥3
5.	How many servings of red meat, hamburger or meat products (ham, sausage, etc.) do you consume per week? (1 serving = 100–150g)	<1
6.	How many servings of butter, margarine, or cream do you consume per week? (1 serving = 12g)	<1
7.	How many sweet/carbonated beverages (soft drinks, cola, bitter, juices without added sugars) do you drink per week?	<1
8.	How much wine (glasses) do you drink per week?	≥7
9.	How many servings of legumes do you consume per week? (1 serving = 150g)	≥3
10.	How many servings of fish or shellfish do you consume per week? (1 serving = 100-150g of fish or 4-5 units or 200g of shellfish)	≥3
11.	How many times per week do you consume commercial sweets or pastries (not homemade), such as cakes, cookies, biscuits, or custard?	<3
12.	How many servings of nuts (including peanuts) do you consume per week? (1 serving = 30g)	≥3
13.	Do you preferentially consume chicken, turkey, or rabbit meat instead of veal, pork, hamburger, or sausage?	Yes
14.	How many times per week do you consume vegetables, pasta, rice, or other dishes seasoned with *sofrito* (sauce made with tomato and onion, leek, or garlic and simmered with olive oil)?	≥2

[1] '0' points if these criteria are not met.

Supplemental Table 2. Association between plasma adiponectin (low vs. high levels using sex-specific cut-off) and the 14-item Mediterranean diet score in the whole population.

		Adiponectin[1]		
**Item**	**Criteria**	**Low**	**High**	***p*[1]**
1. Do you use olive oil as main culinary fat?	Yes	86.3%	83.2%	0.183
2. How much olive oil (tablespoons) do you consume in a given day?	≥4	75.7%	73.0%	0.350
3. How many vegetable servings do you consume per day?	≥2	62.5%	58.3%	0.188
4. How many fruit units (including natural fruit juices) do you consume per day?	≥3	44.7%	50.7%	0.069
5. How many servings of red meat, hamburger or meat products (ham, sausage, etc.) do you consume per week?	<1	59.0%	41.1%	0.195
6. How many servings of butter, margarine, or cream do you consume per week?	<1	90.0%	91.1%	0.586
7. How many sweet/carbonated beverages do you drink per week?	<1	87.0%	89.0%	0.371
8. How much wine (glasses) do you drink per week?	≥7	20.1%	20.1%	0.979
9. How many servings of legumes do you consume per week?	≥3	24.7%	28.9%	0.152
10. How many servings of fish or shellfish do you consume per week?	≥3	49.7%	52.8%	0.361
11. How many times per week do you consume commercial sweets or pastries?	<3	66.4%	61.9%	0.161
12. How many servings of nuts (including peanuts) do you consume per week?	≥3	23.6%	25.7%	0.465
13. Do you preferentially consume chicken, turkey, or rabbit meat instead of veal, pork, hamburger, or sausage?	Yes	75.7%	71.9%	0.196
14. How many times per week do you consume vegetables, pasta, rice, or other dishes seasoned with sofrito?	≥2	56.4%	54.1%	0.480
Total score (high adherence) in points	≥ 9	50.4%	49.3%	0.741

1: A categorical variable for low and high adiponectin concentration was created taking into account sex-specific levels (high≥7.8 (μg/mL) for men, and ≥11.9 (μg/mL) for women). Associations between this variable and adherence to the Mediterranean diet criteria for each item are indicated (as %). P-values correspond to the comparison between high adherence to Mediterranean diet and high adiponectin concentrations for each item and the total score for adherence to Mediterranean diet.

Supplemental Table 3. Association between the rs17300539-*ADIPOQ* promoter polymorphism and metabolic traits. Multivariable adjusted models and gene-diet interactions with adherence to Mediterranean diet.

Metabolic traits	GG[1] Mean±SE	GA Mean±SE	AA Mean±SE	p^2	p^3	*p-int*[4] *Med diet*
BMI (Kg/m^2)	30.20±0.15	30.80±0.29	28.70±0.98	0.627	0.282	0.455
Waist circumference (cm)	102.22±0.43	103.59±0.84	104.84±3.24	0.329	0.121	0.922
HDL-C (mg/dL)	52.73±0.49	51.73±0.99	51.00±2.48	0.580	0.320	0.585
Triglycerides (mg/dL)	130.05±2.45	136.89±5.80	137.11±16.07	0.688	0.267	0.013
Fasting glucose (mg/dL)	120.81±1.44	119.48±2.80	111.73±10.90	0.322	0.344	0.385
Type 2 diabetes (%)	46.6	49.5	47.1	0.716	0.766	0.794

1: Genotype prevalence was 78.4% GG; 19.6% GA and 2.0% AA (n=951). 2: P-value obtained in an unadjusted general linear additive model. 3: P-value obtained in a general linear additive model adjusted for sex, age, diabetes, BMI, physical activity, smoking and adherence to the Mediterranean diet. 4: P-value for the interaction term between the rs17300539 polymorphism and adherence to the Mediterranean diet (high vs. low) in the multivariable regression model including sex, age, diabetes, BMI, physical activity, smoking and adherence to the Mediterranean diet.

Supplemental Figure 1. Manhattan-plot for the SNP-based GWAS on adiponectin concentrations (ln-transformed) in the whole population. A genetic additive model and general linear regression, adjusted for sex, age, diabetes and BMI, were used to estimate p-values, and the top-ranked SNPs were annotated. The red line represents the threshold of suggestive GWAS association ($-\log_{10}(1\times10^{-5})$).

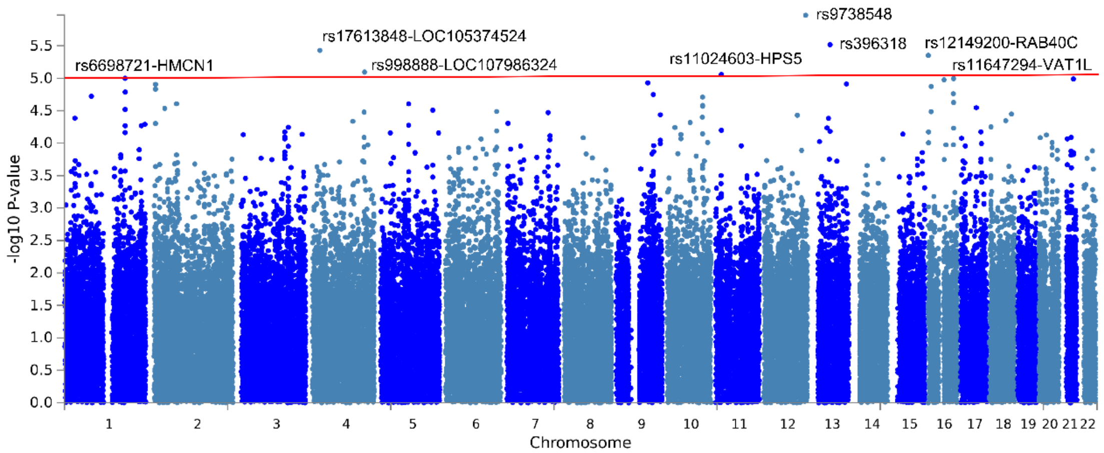

Supplemental Figure 2. QQ-plot for the SNP-based GWAS on adiponectin concentrations (ln-transformed) in the whole population. Model adjusted for sex, age, diabetes and BMI.

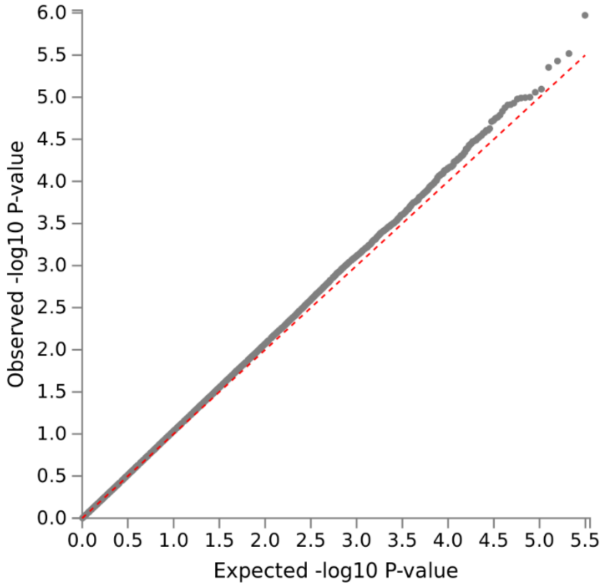

Supplemental Figure 3. Linkage disequilibrium (LD) r^2 plot for the SNPs of the *ADIPOQ* gene (included in the Illumina array and pre-selected)), on chromosome 3.

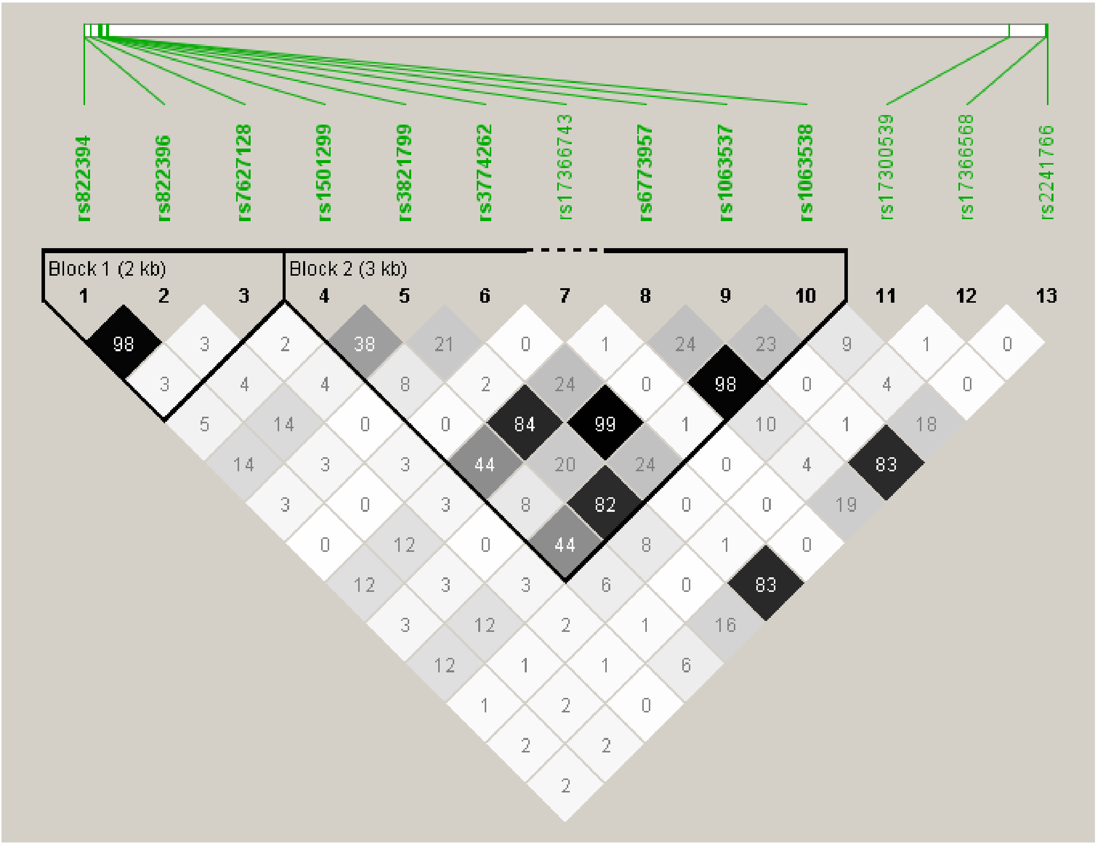

Supplemental Table 4. SNPs of the *ADIPOQ* gene (included in the Illumina array) for adiponectin concentrations (ln-transformed) in the whole population. Model adjusted for sex, age, diabetes and BMI.

Chr	SNP	BP	Beta	*p*	Alleles	MAF	Strand	Gene
3	rs1501299	186571123	0.042	0.080	T	0.300	-	*ADIPOQ*
3	rs6773957	186573705	0.031	0.156	G	0.498	+	*ADIPOQ*
3	rs1063538	186574183	0.029	0.188	C	0.497	+	*ADIPOQ*
3	rs7627128	186568799	0.029	0.320	A	0.179	+	*ADIPOQ*
3	rs3774262	186571814	0.026	0.358	A	0.150	+	*ADIPOQ*
3	rs1063537	186574075	0.026	0.363	T	0.144	+	*ADIPOQ*
3	rs17366743	186572089	0.068	0.394	C	0.011	+	*ADIPOQ*
3	rs3821799	186571486	-0.011	0.600	C	0.458	+	*ADIPOQ*
3	rs822396	186566877	-0.005	0.881	G	0.174	+	*ADIPOQ*
3	rs822394	186566728	-0.003	0.929	A	0.123	+	*ADIPOQ*

BMI: Body mass index. Chr: Chromosome. SNP: Single nucleotide polymorphism. BP: Base position in the chromosome (Homo Sapiens GRCh37.p13 genome build used in Illumina HumanOmniExpress-24 BeadChip). Beta: indicates the effect for the minor allele on adiponectin concentrations (ln-trasformed). *p:* P-value obtained in the multivariable linear regression model adjusted for sex, age, diabetes and BMI for each SNP using a genetic additive model. MAF: minor allele frequency.

Supplemental Table 5. Top-ranked SNPs of the *CDH13* gene for adiponectin concentrations (ln-transformed) in the whole population. Model adjusted for sex, age, diabetes and BMI. The 20 most significant SNPs from the total of 72 *CDH13* SNPs included in the Illumina array are presented.

Chr	SNP	BP	Beta	*p*	Alleles	MAF	Strand	Gene
16	**rs4782726**	82701333	-0.068	**0.016**	A	0.198	+	*CDH13*
16	rs4508407	83145350	0.038	0.102	G	0.493	+	*CDH13*
16	rs10514564	82741256	0.038	0.102	G	0.424	-	*CDH13*
16	rs11859453	83623678	-0.034	0.128	A	0.432	+	*CDH13*
16	rs16960234	83356008	0.057	0.137	C	0.050	+	*CDH13*
16	rs1433157	82678223	-0.032	0.154	C	0.478	-	*CDH13*
16	rs17284390	83397287	0.047	0.186	G	0.044	+	*CDH13*
16	rs10514569	83476104	0.064	0.209	G	0.039	-	*CDH13*
16	rs7500599	83091869	0.027	0.209	G	0.464	+	*CDH13*
16	rs7198915	82706126	-0.031	0.238	C	0.202	+	*CDH13*
16	rs1862830	83303147	-0.025	0.240	G	0.484	+	*CDH13*
16	rs10514585	83284338	-0.029	0.241	A	0.292	-	*CDH13*
16	rs8055389	82718030	-0.032	0.289	G	0.107	+	*CDH13*
16	rs2306907	83520078	0.027	0.299	A	0.183	-	*CDH13*
16	rs1862831	83323634	0.024	0.309	G	0.382	+	*CDH13*
16	rs8060632	83625563	0.023	0.318	A	0.279	+	*CDH13*
16	rs7184058	83270582	0.025	0.343	A	0.140	+	*CDH13*
16	rs7200573	83621967	0.023	0.344	A	0.282	+	*CDH13*
16	rs8054845	83487316	-0.021	0.349	G	0.474	+	*CDH13*
16	rs8055236	83212398	-0.025	0.352	T	0.262	+	*CDH13*

BMI: Body mass index. Chr: Chromosome. SNP: Single nucleotide polymorphism. BP: Base position in the chromosome (Homo Sapiens GRCh37.p13 genome build used in Illumina HumanOmniExpress-24 BeadChip). Beta: indicates the effect for the minor allele on adiponectin concentrations (ln-trasformed). *p:* P-value obtained in the multivariable linear regression model adjusted for sex, age, diabetes and BMI for each SNP using a genetic additive model. MAF: minor allele frequency.

Supplemental Table 6. Top-ranked SNPs of the GWAS for adiponectin concentrations (ln-transformed) in men and women. Model adjusted for age, diabetes and BMI.

A: Men

Chr	SNP	BP	Beta	*p*	Alleles	MAF	Strand	Gene
14	rs4903705	78555941	0.222	$1.01×10^{-6}$	T	0.163	+	*NRXN3*
2	rs7560042	154456164	0.196	$1.35×10^{-6}$	A	0.237	+	intergenic
1	rs13375867	100575933	0.174	$2.02×10^{-6}$	A	0.350	+	*SASS6*
14	rs10483889	78557505	0.212	$3.04×10^{-6}$	G	0.108	+	*NRXN3*
1	rs5876	100547994	0.169	$4.13×10^{-6}$	C	0.352	+	*MFSD14A*
2	rs10211067	98703659	-0.166	$4.64×10^{-6}$	T	0.423	+	*VWA3B*
1	rs12406605	100502690	0.168	$4.74×10^{-6}$	C	0.345	+	*MFSD14A*
4	rs17045969	166030858	-0.372	$5.42×10^{-6}$	T	0.118	+	*TMEM192*
2	rs10197706	154472066	0.182	$5.81×10^{-6}$	C	0.200	+	intergenic
1	rs10914455	32085637	0.369	$6.07×10^{-6}$	G	0.086	+	*HCRTR1*
6	rs6923822	128839470	-0.251	$6.55×10^{-6}$	A	0.156	+	*PTPRK*
13	rs9315422	37055511	0.180	$7.93×10^{-6}$	T	0.277	+	intergenic
1	rs11166413	100656494	0.164	$8.63×10^{-6}$	G	0.264	+	*DBT*
22	rs5752494	27523485	0.170	$9.00×10^{-6}$	C	0.396	+	intergenic

B: Women

Chr	SNP	BP	Beta	*p*	Alleles	MAF	Strand	Gene
12	rs9989048	79305654	0.165	$1.54×10^{-7}$	T	0.278	+	*SYT1*
12	rs4529943	79310416	0.164	$1.93×10^{-7}$	A	0.251	+	*SYT1*
11	rs601727	100486632	0.145	$2.14×10^{-7}$	C	0.411	+	intergenic
15	rs1667394	28530182	-0.134	$2.48×10^{-6}$	T	0.380	-	*HERC2*
10	rs7894696	80200607	0.155	$3.26×10^{-6}$	T	0.232	+	*LOC107984245*
15	rs916977	28513364	-0.130	$5.42×10^{-6}$	C	0.389	-	*HERC2*
7	rs3757791	127233068	0.384	$7.12×10^{-6}$	T	0.111	-	*FSCN3*
7	rs3757790	127233279	0.384	$7.12×10^{-6}$	T	0.111	-	*FSCN3*
7	rs16131	24329837	-0.176	$7.15×10^{-6}$	C	0.087	-	*NPY*
13	rs11618218	51689586	0.147	$7.65×10^{-6}$	T	0.192	+	*LINC00371*
12	rs10778419	106060102	0.140	$8.63×10^{-6}$	A	0.145	+	intergenic
11	rs11024603	18306399	0.153	$1.03×10^{-5}$	A	0.228	+	*HPS5*
4	rs17613848	23485012	-0.151	$1.05×10^{-5}$	A	0.135	+	*LOC105374524*
7	rs13223753	24311728	-0.171	$1.07×10^{-5}$	C	0.083	+	*LOC107986777*
6	rs1283468	70038147	0.166	$1.11×10^{-5}$	A	0.180	+	*ADGRB3*

CHR: Chromosome. SNP: Single nucleotide polymorphism. BP: Base position in the chromosome (Homo Sapiens GRCh37.p13 genome build used in Illumina HumanOmniExpress-24 BeadChip). BETA: indicates the effect for the minor allele on adiponectin concentrations (ln-trasformed). *p:* P-value obtained in the multivariable linear regression model adjusted for age, diabetes and BMI for each SNP using a genetic additive model. MAF: minor allele frequency.

Supplemental Figure 4. Linkage disequilibrium (LD) regional plot for the SNP rs5876 (marked as sentinel SNP), located in the hippocampus abundant transcript 1 (*HIAT*) gene, also known as *MFSD14A* gene, on chromosome 1 in the GWAS, for adiponectin concentrations in Men. Each SNP are colored based on r^2.

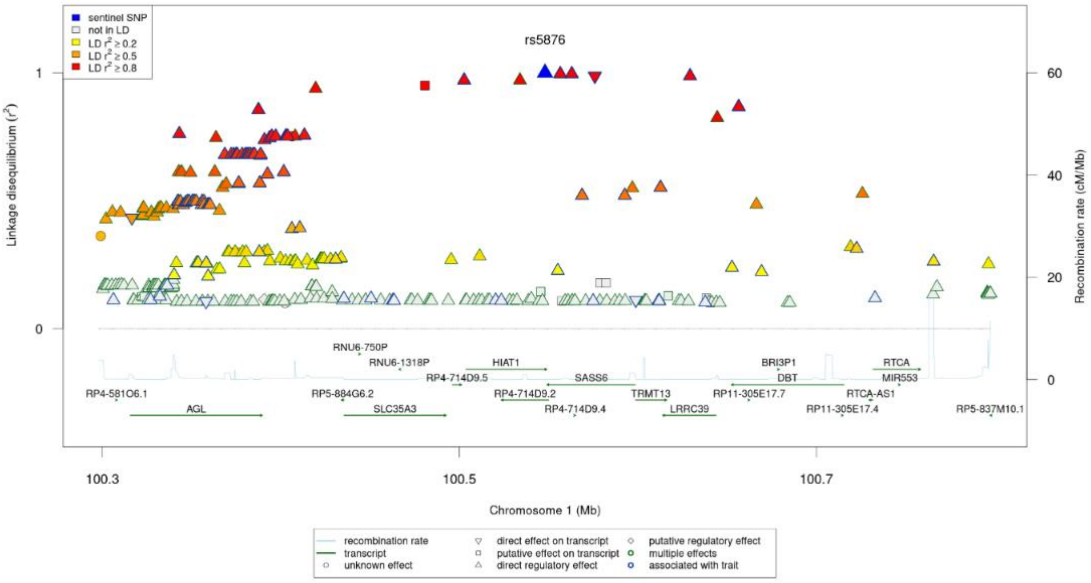

Supplemental Figure 5. Linkage disequilibrium (LD) regional plot for the SNP rs9989048-Synaptotagmin 1 (*SYT1*) (marked as sentinel SNP), on chromosome 12, and the regional plot obtained in the GWAS for adiponectin concentrations (ln-transformed) in women. Each SNP are colored based on r^2.

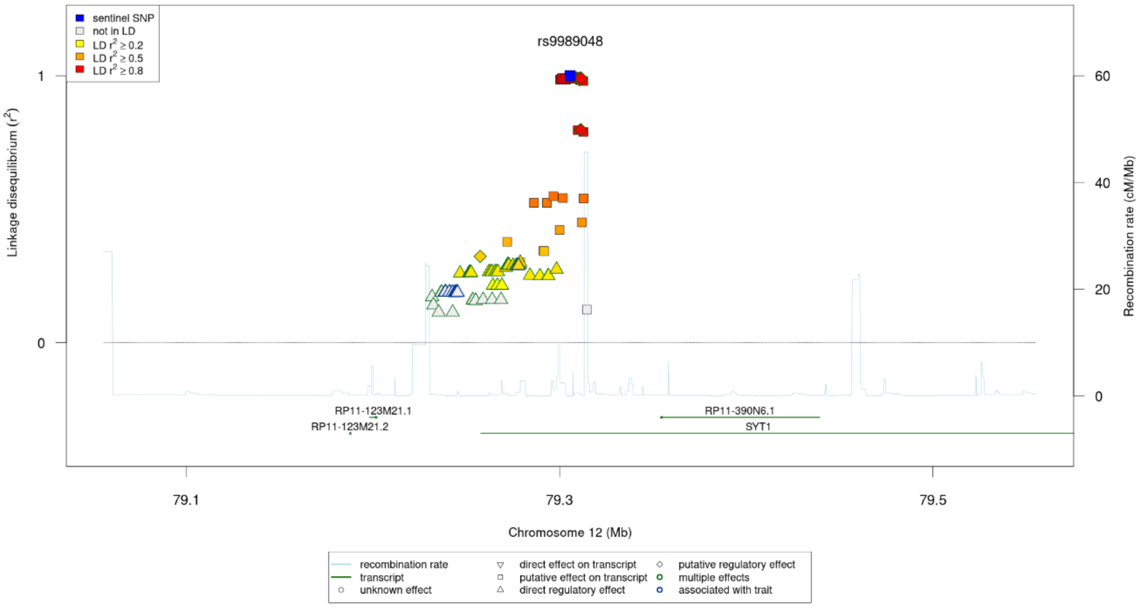

Supplemental Figure 6. Regional plot for the sentinel SNP rs7616406-*CAND2*, on chromosome 3. Each SNP are colored based on r^2.

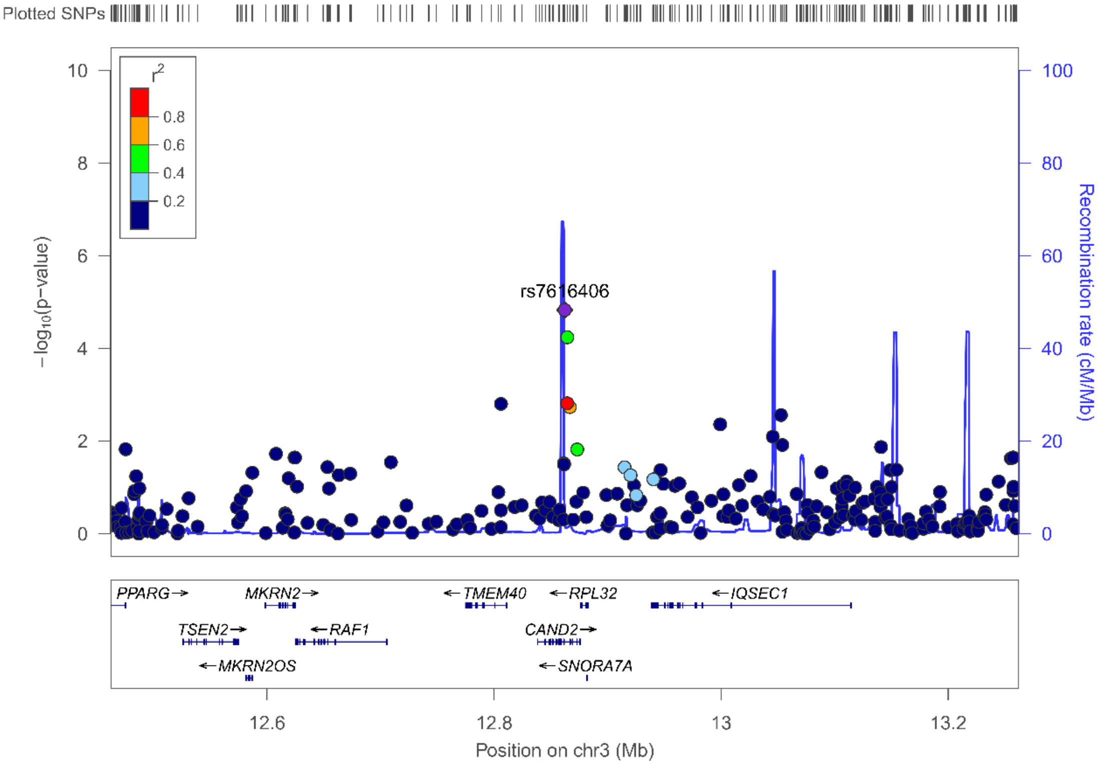

Supplemental Figure 7. Linkage disequilibrium (LD) plot for the SNP rs17249128-*LOC101927334* (marked as sentinel SNP), on chromosome 16, in the GWAS for the gene-Mediterranean diet interactions on plasma adiponectin concentrations (ln-transformed). Each SNP are colored based on r^2.

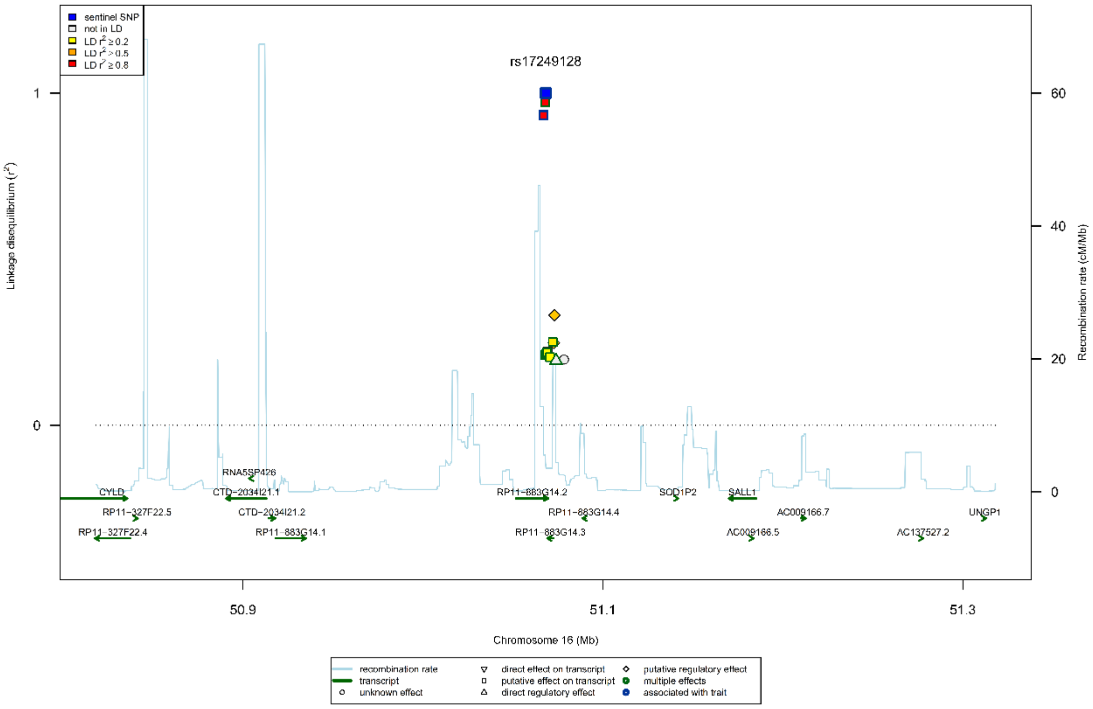

Supplement: Supplementary file 1 [file nutrients-13-00541-s001.pdf]
